# How do China’s lockdown and post-COVID-19 stimuli impact carbon emissions and economic output? Retrospective estimates and prospective trajectories

**DOI:** 10.1016/j.isci.2022.104328

**Published:** 2022-04-30

**Authors:** Shuai Shao, Chang Wang, Kuo Feng, Yue Guo, Fan Feng, Yuli Shan, Jing Meng, Shiyi Chen

**Affiliations:** 1 School of Business, East China University of Science and Technology, Shanghai 200237, China; 2 School of Economics, Fudan University, Shanghai 200433, China; 3 The Whitney and Betty MacMillan Center for International and Area Studies, Yale University, New Haven 06511, USA; 4 School of Economics, Zhejiang University of Finance & Economics, Hangzhou 310018, China; 5 Zhejiang Institute of “Eight-Eight” Strategies, Zhejiang University of Finance & Economics, Hangzhou 310018, China; 6 Key Laboratory of Regional Sustainable Development Modeling, Institute of Geographic Sciences and Natural Resources Research, Chinese Academy of Sciences, Beijing 100101, China; 7 College of Resources and Environment, University of Chinese Academy of Sciences, Beijing 100049, China; 8 Warwick Business School, University of Warwick, Coventry CV4 7AL, UK; 9 Renmin Business School, Renmin University of China, Beijing 100872, China; 10 Integrated Research on Energy, Environment and Society (IREES), Energy and Sustainability Research Institute Groningen, University of Groningen, Groningen 9747 AG, the Netherlands; 11 School of Geography, Earth and Environmental Sciences, University of Birmingham, Birmingham B15 2TT, UK; 12 The Bartlett School of Sustainable Construction, University College London, London WC1E 7HB, UK; 13 Anhui University, Hefei 230601, China

**Keywords:** energy policy, energy sustainability, economics

## Abstract

This paper develops a multi-sector and multi-factor structural gravity model that allows an analytical and quantitative decomposition of the emission and output changes into composition and technique effects. We find that the negative production shock of China’s containment policy propagates globally via supply chains, with the carbon-intensive sectors experiencing the greatest carbon emission shocks. We further reveal that China’s current stimulus package in 2021–2025 is consistent with China’s emission intensity-reduction goals for 2025, but further efforts are required to meet China’s carbon emissions-peaking target in 2030 and Cancun 2°C goal. Short-term changes in carbon emissions resulting from lockdowns and initial fiscal stimuli in “economic rescue” period have minor long-term effects, whereas the transitional direction of future fiscal stimulus exerts more predominant impact on long-term carbon emissions. The efficiency improvement effects are more important than the sectoral structure effects of the fiscal stimulus in achieving greener economic growth.

## Highlights

• Negative shocks of China’s lockdown propagate globally via supply chains
• China’s current stimulus packages alone are insufficient for CO_2_ peaking target and 2°C goal
• Lockdowns and initial fiscal stimulus have minor long-term effects on CO_2_
• Scale and efficiency effects drive green growth more than sectoral structure effects

## Graphical abstract

**Figure:**
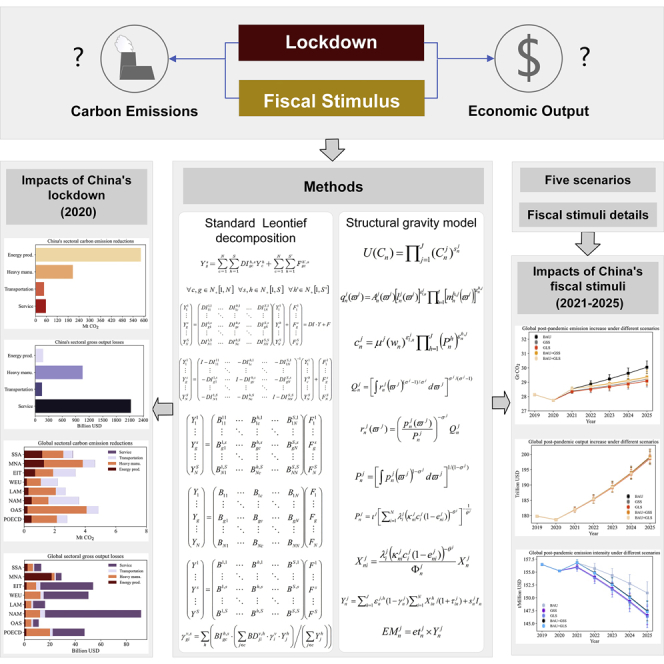

## Introduction

**Figure 1:**
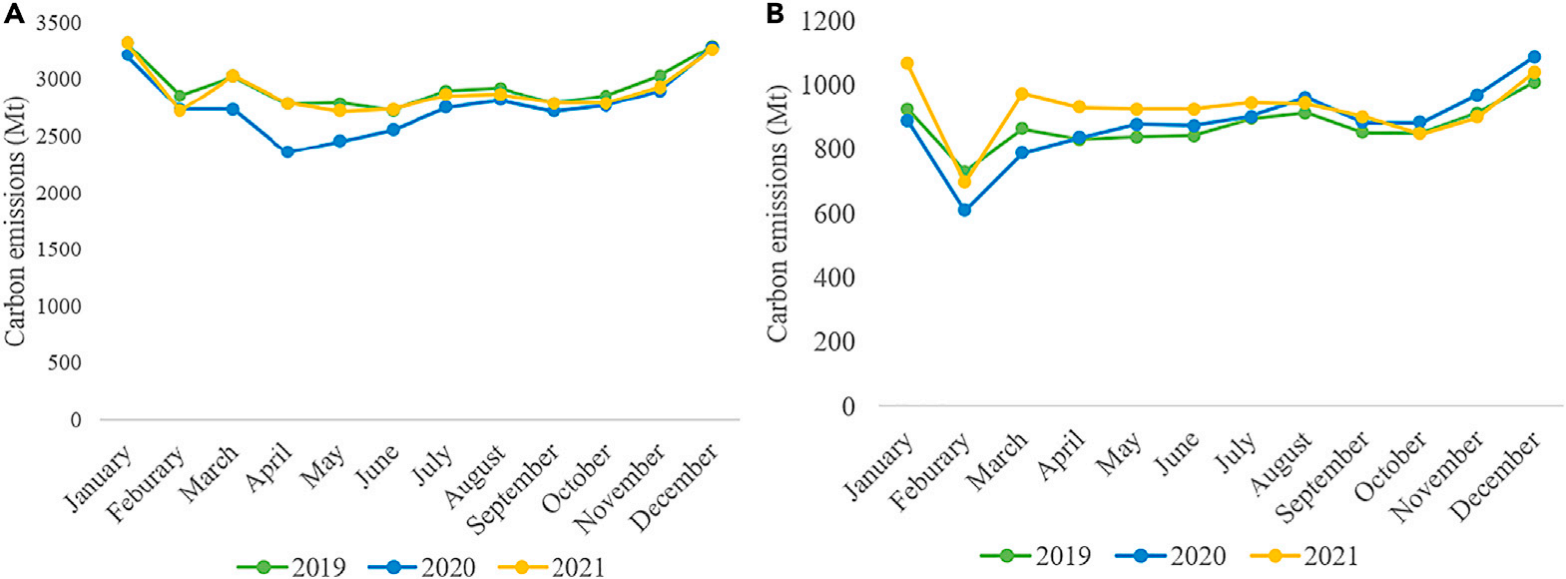
Trends in carbon emissions during 2019–2021 (A) Trends in global carbon emissions during 2019–2021; (B) Trends in China’s carbon emissions during 2019–2021.

The pandemic is expected to cause the largest reduction in annual carbon emissions (short for carbon dioxide (CO_2_) emissions) ever recorded (Hepburn et al., 2020). According to the data from Carbon Monitor, global carbon emissions drop by 5.7% in 2020 compared to 2019, as shown by Figure 1A (Liu et al., 2020b). As the earliest outbreak center of the COVID-19, China has become one of the first major economies to implement stringent containment measures, launch fiscal stimulus packages, and gain positive economic growth (Inoue and Todo, 2019; IMF, 2020a; WHO, 2020). However, the quick recovery accompanied by intensive fiscal stimulus measures may result in a carbon emission surge. In fact, as Figure 1B shows, China’s monthly average carbon emissions in 2021 have rebounded and reached above its pre-pandemic level in 2019 by 5.1%, surpassing China’s emission drop in 2020. The global economy, on the other hand, keeps plagued by fluctuations in COVID-19 cases, with carbon emissions rising up in 2021 but not reaching the pre-pandemic level. Therefore, China’s post-COVID-19 stimuli, especially the long-term shift of China’s stimuli toward fossil-fueled growth or green growth after the tentative return-to-normal steps, may add uncertainties to future carbon emission mitigation pathways.

A few studies have investigated the socioeconomic impacts of the COVID-19 containment measures (Duan et al., 2020; McKibbin and Fernando, 2021; Josephson et al., 2021; Ke and Hsiao, 2021; Tan et al., 2021; Wan et al., 2021). For example, Guan et al. (2020) developed a disaster footprint model to quantitatively assess the worldwide economic footprints under different containment scenarios. By integrating the interaction between containment policy decisions and COVID-19 infection rates, Eichenbaum et al. (2021) studied the appropriate containment policy designs for achieving the dual purpose of lessening economic recession and COVID infections. Some studies also discussed the propagation effect of a country’s containment policy to other economies via the supply chains (Duan et al., 2021; Ivanov, 2020; Pan et al., 2021; Pichler and Farmer, 2021). A potential benefit of the containment measures is the reduction in carbon emissions resulting from forced shutdowns of production facility (Forster et al., 2020; Friedlingstein et al., 2020; Liu et al., 2020a; Turner et al., 2020; Zheng et al., 2020; Schulte-Fischedick et al., 2021). One of the earliest estimates of carbon emission reduction is from Myllyvirta (2020), with the estimated carbon emission reduction of around 2000 million metric tonnes (Mt) in 2020. IEA estimated a -5% decline in global emissions in the first quarter of 2020 compared to the same period in 2019 (IEA, 2020). In addition, Liu et al. (2020c) used a high temporal resolution emission dataset to estimate a decrease of 4.2% in global carbon emissions in the first quarter of 2020. Similarly, Le Quéré et al. (2020) combined information on both production activities and government policies and proposed a daily global carbon emission decrease of 17% in April 2020.

With the COVID-19 pandemic under control in more and more regions, existing studies started to focus on the appropriate design of economic stimulus plans in the post-COVID-19 period. A strand of studies focused on the macroeconomic effects of fiscal stimuli (Guerrieri et al., 2020; Porsse et al., 2020; Liu et al., 2021). Some researchers further discussed the climate impact of fiscal stimuli (IEA, 2020; Lahcen et al., 2020; Pollitt et al., 2021; Tian et al., 2022). For example, Tian et al. (2022) reviewed the post-COVID-19 economic recovery stimuli in major economies, and qualitatively analyzed the impacts of economic stimuli on energy transition. Shan et al. (2021) used an adaptive input-output model to analyze the worldwide carbon impacts of different containment and recovery strategies. Hepburn et al. (2020) and Batini et al. (2021) noted that policies with high potential for both economic recovery and emission mitigation are achievable, as the output multiplier on climate-friendly sectors is higher than non-eco-friendly sectors.

However, most of the previous studies analyzed the impacts of the containment policies on carbon emissions or economic output, the emission and economic impacts are not linked together. The key question of achieving climate-friendly economic recovery when designing recovery packages remains unsolved (Kuzemko et al., 2020). To fill this gap, our study takes China as an example and focuses on the impact of its containment policies on domestic and global emissions and economic output at the sector level, rendering the discussion about the green transition opportunities associated with carbon-intensive production disruption. In terms of the impacts of post-COVID-19 fiscal stimuli, owing to data limitation and unclear economic recovery plans, existing literature generally either analyzed the green growth effect of fiscal stimuli policies based on hypothetical and self-designed stimulus policies or at aggregated sector level with low granularity. As the largest developing country, China is an ideal study object because of the availability of input-output and emission data, quick production resumption after the pandemic, and the transparency of economic stimulus packages. Second, existing studies usually investigated the overall impact of the COVID-19 pandemic, instead of focusing on the impacts of the containment policy and fiscal measures in a specific policy setting and quantifying how the impacts propagated via the global supply chains. Last but not the least, existing literature did not generally consider the policy focus changes, and instead assumed the same set of policies through the coming years. In reality, however, stimulus policies tend to focus on urgent economic recovery needs in the first year of implementation, and transit to green or brown-based patterns later on. Our research, instead, designs future scenarios based on China’s announced sector-level fiscal stimuli, and quantitatively assesses the domestic and propagation effects on carbon emissions and economic output, with the aid of a structural gravity model. Our scenario set encompasses five scenarios, which illustrates China’s current announced fiscal stimuli, the greener fiscal stimuli, and the combination of current announced and greener fiscal stimuli. In particular, the combination of current announced and greener fiscal stimuli depicts the transition of China’s fiscal stimuli from economic recovery stage to green-based growth.

In sum, by using a multi-sector gravity model considering the nested relationship between domestic and international input-output tables, we quantitatively assess both the carbon and economic impacts of China’s containment policy across nine aggregated economies, with each economy divided into 22 sectors to illustrate how the current pandemic-related carbon emission reductions and output losses are distributed along the global supply chains in 2020. Furthermore, we design several scenarios on China’s post-pandemic fiscal stimulus packages to understand China’s midterm (i.e., 2021–2025) carbon emissions and output growth under the interaction of increased fiscal investment and efficiency enhancement.

Our investigation into the specific policy setting in China enables us to provide policy recommendations more accordingly for the post-COVID-19 economic recovery while minimizing adverse environmental impacts. Meanwhile, it should be noted that because of uncertainties of future actual policies and the scope of our data, the goal of this study is not to comprehensively assess the true midterm impacts of the COVID-19 pandemic. On the contrary, we aim to identify how effective containment measures and different fiscal stimulus packages exert heterogeneous impacts on carbon emissions and economic output through global supply chains and highlight the significance of fiscal stimulus measures on climate change mitigation and economic recovery.

## Results

### Global impacts of China’s containment policy

**Figure 2:**
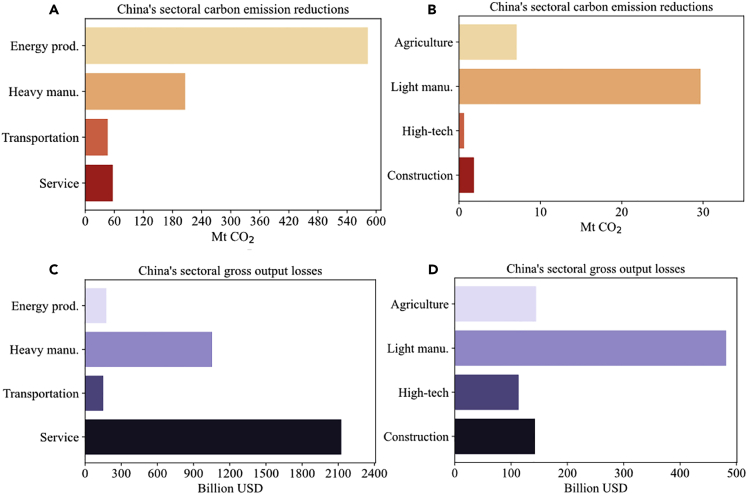
China’s sectoral carbon emission reductions and gross output losses caused by China’s containment policy in the first half of 2020 (A) China’s sectoral carbon emission reductions in energy production, heavy manufacturing, transportation, and service sectors; (B) China’s sectoral carbon emission reductions in other sectors; (C) China’s sectoral gross output losses in energy production, heavy manufacturing, transportation, and service sectors; (D) China’s sectoral gross output losses in other sectors.

**Figure 3:**
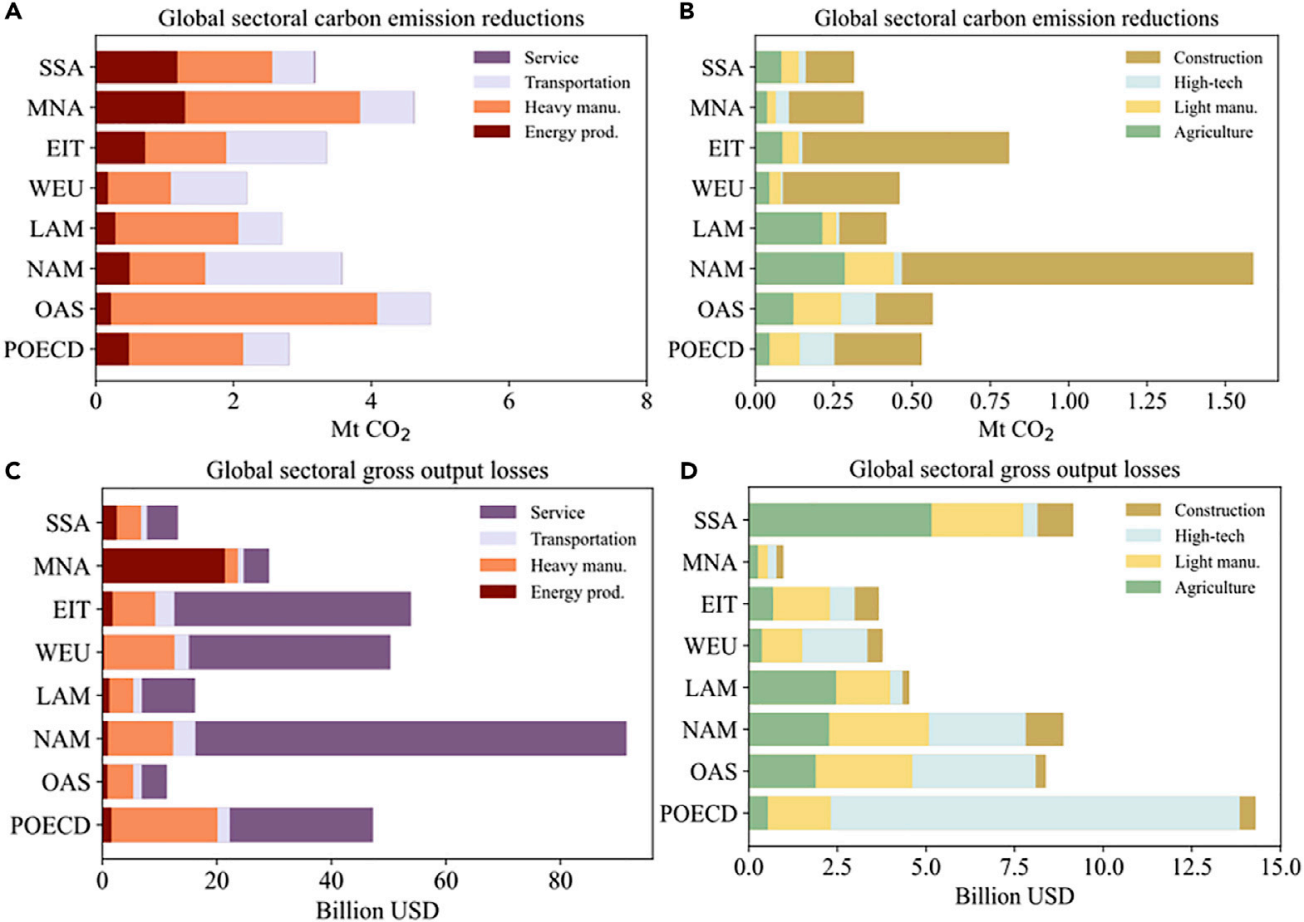
Global sectoral carbon emission reductions and gross output losses outside China caused by China’s containment policy in the first half of 2020 SSA, MNA, EIT, WEU, LAM NAM, OAS, and POECD represent Sub-Saharan Africa, Middle East and North Africa, economies in transition (Eastern Europe and former Soviet Union), Western Europe, Latin America and Caribbean, North America (USA, Canada), developing regions in Asia and Pacific, and developed regions in Asia and Pacific, respectively. (A) Global sectoral carbon emission reductions in service, transportation, heavy manufacturing, and energy production sectors; (B) Global sectoral carbon emission reductions in other sectors; (C) Global sectoral gross output losses in service, transportation, heavy manufacturing, and energy production sectors; (D) Global sectoral gross output losses in other sectors.

Figures 2 and 3 present direct (because of China’s containment policy) and global propagation effects of China’s containment policy on sectoral carbon emissions and economic output in world regions via global supply chains (see Data S1 for the categorization of sectors), respectively. The strict restrictions of industrial production and traveling interrupt production and transportation in China, generating a COVID-19-linked immediate carbon emission reduction of 931.2 Mt within China in the first half of 2020, which is 11.0% of the total China’s carbon emissions of 2019. Meanwhile, China’s containment policy leads to a negative economic shock of 4.4 trillion USD in the first half of 2020, accounting for 11.6% of China’s pre-pandemic gross output. We compare our emission-reduction estimate with Le Quéré et al. (2020) and Liu et al. (2020c), who used real-time activity data to find that China’s pandemic-related carbon emission reductions are -108 to -394 Mt and -187.2 Mt, respectively. The main reason for such a gap in estimation is that we provide the counterfactual estimates, i.e., the difference between the carbon emissions in 2020 without the COVID-19 and the actual carbon emissions in 2020. Their estimates, on the other hand, are based on the comparison between actual carbon emissions in 2019 and 2020, in absence of accounting for the pre-pandemic economic growth trend. (We also use our model to estimate the gap between China’s actual emissions in 2019 and 2020. The result is 114.2 Mt, which is quite similar with the estimates of Le Quéré et al. (2020) and Liu et al. (2020c)). Moreover, the estimates based on real-time data only consider key sectors’ activity changes, such as energy production and heavy manufacturing sectors. Our estimate, instead, is based on activity changes in all the economic sectors. We also compare our output-loss estimates to the output growth rate estimate of Tan et al. (2021). Combining the CGE model with a hypothetical scenario, Tan et al. (2021) indicate that the counterfactual loss rate of total output in 2020 is -8.5%, which is similar to our results (-11.6%). The difference could be because of the fact that the estimate of Tan et al. (2021) is based on the aspects of the direct sectoral output shock, international trade, and labor force, while our model considers both direct shocks and sectoral input-output linkages.

In addition, we find the impact of the containment policy is highly localized, with China accounting for 96.6% carbon emission reductions and 92.3% gross output losses during the first half of year 2020. Figure 2 illustrates China’s sector-wise changes in carbon emissions and economic loss. An essential insight from the estimates is that the impacts of the containment measures implemented in one country are worldwide, even spreading to countries with no COVID-19 cases and containment measures.

Figure 3 presents the estimated propagation effects of China’s containment policy on global carbon emissions and economic output in world regions (China excluded) via global supply chains (see Data S1 and S2 for the categorization of sectors and world regions, respectively). All the world regions (China excluded) are influenced by China’s containment policy, leading to reductions in carbon emissions and global output of 32.3 Mt (0.1% of global emissions) and 366.4 billion USD (0.2% of global output), respectively. North America and developing regions in the Asia and Pacific (i.e., NAM and OAS in Figure 3) experience greater emission reduction, because the supply chain trade is relatively more frequent within these regions (Maurer, 2017). Because of the relatively higher emission intensity, the developing regions in Asia and Pacific (OAS), Latin America and Caribbean (LAM), Middle East and North Africa (MNA), and Sub-Saharan Africa (SSA) are generating relatively higher emission reduction than economic loss.

The pandemic control mainly reduces the worldwide carbon emissions and economic output in energy production, heavy manufacturing, transportation, and service, and thus can be harvested as an opportunity to reduce the capacity of carbon-intensive industries, leading to worldwide carbon emission mitigation. Subsectors of chemical products (-54.0 Mt; -342.7 billion USD), metal smelting (-51.1 Mt; -265.8 billion USD), and transportation (-54.4 Mt; -167.8 billion USD) witness significant declines in both carbon emissions and economic output. The subsectors that are relatively more crucial for economic recovery and less responsible for climate change are service (-59.9 Mt; -2325.6 billion USD), electronic and telecommunications equipment (-0.6 Mt; -113.7 billion USD) and clothing, leather, fur, etc. (-0.5 Mt; -32.4 billion USD), indicating that economic stimulus packages can focus on boosting these low-carbon subsectors. The centralized emission shocks in energy production and heavy manufacturing imply that the outbreak of the COVID-19 can be viewed as an opportunity to achieve structural adjustment toward low-carbon and high-quality economic growth. The economic output shock centers more in service, which takes up about 50% of pandemic-related gross economic output shock. Subsectors that are most negatively impacted may include tourism, wholesale and retail trade, accommodation, and the catering sector, which generally experience greater revenue loss during economic crises. It should also be noted that the carbon emissions and economic output of energy production and heavy manufacturing sectors in developing countries are more severely impacted. For many developing countries, industrialization is an engine of poverty eradication that is highly dependent on global upstream suppliers. Thus, as lockdowns become a continuing global action, developing countries could bear more severe and long-lasting socioeconomic consequences, because they lack resources to cope with the supply chain risks, unemployment, and the collapse of global demand (Montalbano, 2011).

### Increases in output and emissions from fiscal stimulus packages

**Figure 4:**
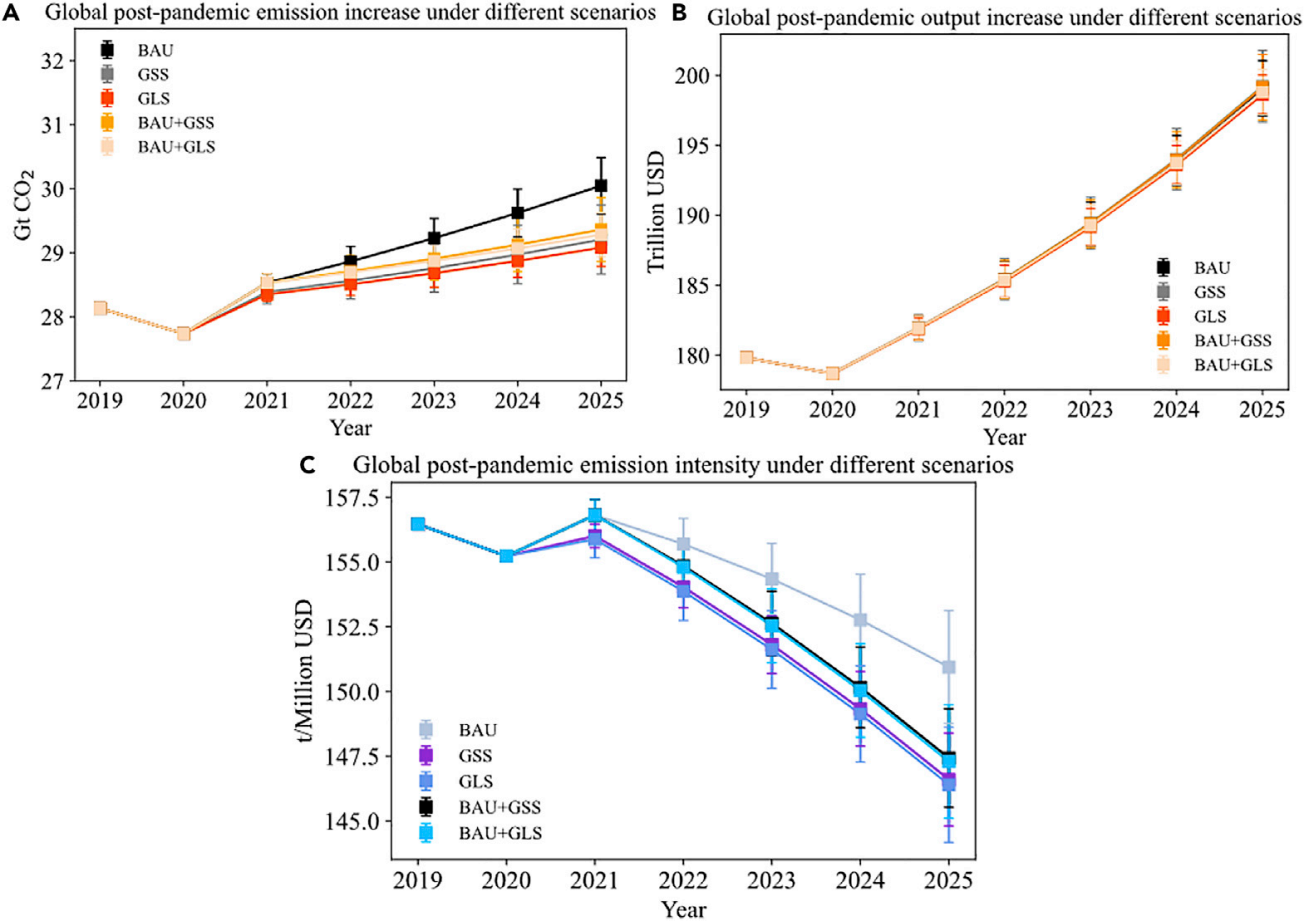
Global post-pandemic output and emission growth under various scenarios of fiscal stimuli BAU, GSS, GLS, BAU + GSS, and BAU + GLS denote business-*as*-usual, green stimulus, green lifestyle, business-*as*-usual to green stimulus, and business-*as*-usual to green lifestyle scenarios, respectively. The ranges of future values are represented by confidential intervals, whereas the median levels are represented by dots. (A) Global post-pandemic emission increases under different scenarios; (B) Global post-pandemic output increases under different scenarios; (C) Global post-pandemic emission intensity under different scenarios.

In this section, we focus on the emission impacts of China’s post-pandemic fiscal counter measures on carbon emissions and economic output. In detail, we use the five scenarios to investigate the impacts of China’s supply chain recovery on global economic output and carbon emissions. The specific scenario settings are described in the method details section. China announced a fiscal stimulus package of 6.5% of national GDP in 2020, which is used to design the business-*as*-usual (BAU) scenario in this section. We also deviate from current stimulus to light manufacturing, high-tech, and service sectors, and we assume greater decrease in carbon emission intensity to construct green stimulus (GSS) and green lifestyle (GLS) scenarios. In the initial post-COVID-19 “economic rescue” period, fiscal stimuli are highly likely to be “colorless” or brown-based, as the major purposes of economic recovery are reducing unemployment and resuming normal business operation. For example, China approved three new coal-fired plants of nearly 10 GW (GW) in March, 2020, roughly equal to the total amount approved for last year (Farand, 2020). However, stimulus policy focus may shift toward climate change mitigation later on, and the climate-friendly policy choices have the potential to drive a long-term downturn in carbon emissions (Hepburn et al., 2020). Based on this understanding, we also design two other scenarios by using the combination of BAU (in the year 2021) and GSS/GLS (in the year 2022–2025), namely BAU + GSS and BAU + GLS. Figure 4B shows the future output during the period of 2021–2025 under different stimulus measures. We find that the current stimulus package of China is enough for output recovery to the pre-pandemic level in 2021. Under the BAU scenario, the global output in 2021 will be 1.2% (0.7%–1.7%) higher than the pre-pandemic level in 2019, mounting to 181.9 trillion USD (181.1 trillion USD to 182.8 trillion USD). In 2025, fiscal stimulus packages with different structures but same scale will generate substantial but similar gains in economic output, and the global output level in 2025 will range from 196.7 trillion to 201.8 trillion USD. This result indicates the structure of fiscal stimulus packages does not bring about much change in output, whereas the scale of stimulus packages is more important.

On the contrary, Figure 4A shows that the different levels of future carbon emissions are observed in the five scenarios. The BAU recovery scenario assumes a concentrated stimulus on construction sectors, generating high embodied emissions in upstream demands from carbon-intensive raw materials and electricity (Su and Thomson, 2016). Meanwhile, more fiscal resources are allocated to output expansion instead of efficiency enhancement, resulting in the largest increase in emissions. Under the BAU recovery scenario, the global carbon emissions will increase to around 30 Gt (29.6 Gt to 30.5 Gt) in 2025. The GSS and the GLS scenarios both assume a structural reduction in China’s emission intensity and more financial funds flowing to clean sectors. The consequences of the greenest stimulus packages (as specified in GLS scenario) are decelerated emission growth trends after 2021, stabilizing around 29.1 Gt (28.8 Gt to 29.4 Gt). However, the climate impacts of the BAU + GSS and BAU + GLS scenarios are quite similar to GSS and GLS scenarios. In 2021, BAU + GSS and BAU + GLS scenarios follow the same scenario settings as BAU scenario, and push the global carbon emissions to 28.5 Gt (28.4 Gt to 28.7 Gt). In the later years, aligning with GSS and GLS scenarios, greener stimuli are assumed, and the total carbon emissions start to deviate from the level of BAU and converge to scenarios with lower emission levels, i.e., GSS and GLS. In 2025, the median emissions in BAU + GSS and BAU + GLS are 29.4 and 29.3 Gt, only 0.2 Gt higher than GSS and GLS scenarios, respectively.

Nevertheless, the five stimulus packages all generate the carbon emission level of more than 28 Gt in 2025, and none can achieve lower emissions than the upper bound of the 2°C Cancun climate goal (around 27 Gt carbon emissions from economic sectors) in 2025 (Climate Action Tracker, 2019). Additional efforts, such as deep decarbonization of global energy systems and strong decoupling effects of Global North countries, are urgently required.

From Figure 4C, we find that the COVID-19 containment policy will generate a 0.8% decrease in emission intensity in 2020 because of primary disruptions in carbon-intensive sectors. The decrease will be offset immediately in 2021 because of stimulus policies in the BAU scenario. It is noteworthy that the trends of future global emission intensity are analogous under scenarios with the same assumptions of emission intensity change but different assumptions of fiscal stimulus package structure. For example, the 2025 global emission intensity under the BAU scenario is around 151 t/10^6^ USD (147.3 t/10^6^ USD to 154.7 t/10^6^ USD), and the emission intensities under the GSS and GLS scenarios are both around 146 t/10^6^ USD (142.1 t/10^6^ USD to 151.2 t/10^6^ USD).

Table 1 lists the corresponding results for China’s post-pandemic output and emission growth under various scenarios of fiscal stimuli. Our calculation also manifests China’s emission intensity during 2021–2025 will most likely decline by 18.5%, 23.5%, 23.5%, 22.8%, and 23.6% under the BAU, GSS, GLS, BAU + GSS, and BAU + GLS scenarios, respectively. In the 14th Five-Year Plan period (2021–2025), China sets the target of the decline in emission intensity by 18%. Therefore, China’s current fiscal stimulus policy is enough to meet the 14th Five-Year Plan, in absence of considering other carbon emission reduction forces, such as structural transformation and technology breakthrough. Given the rising future carbon emissions in all the five scenarios, additional efforts are still required to peak China’s emissions before 2030. In sum, our results show that among these five scenarios, the patterns of post-pandemic output increase are similar, whereas the trends of emissions and emission intensity vary, with the greenest packages achieving a flattening trend of future carbon emission increase.

**Table 1:** China’s post-pandemic output and emission growth under various scenarios of fiscal stimuli

Year	Scenarios	Carbon emissions (Gt)	Economic output (Trillion USD)	Emission intensity (t/Million USD)
2021	BAU	9.3 [9.2, 9.4]	43.3 [43.0, 43.6]	214.8 [211.0, 218.6]
	GSS	9.2 [9.0, 9.4]	43.4 [42.8, 44.0]	212.0 [204.5 ,219.6]
	GLS	9.1 [9.0, 9.2]	43.1 [42.6, 43.6]	211.1 [206.4 ,216.0]
	BAU + GSS	9.3 [9.2, 9.4]	43.4 [43.0, 43.8]	214.3 [210.0, 218.6]
	BAU + GLS	9.3 [9.2, 9.4]	43.4 [43.1, 43.7]	214.3 [210.5, 218.1]
2025	BAU	11.4 [11.0, 11.8]	65.1 [64.1, 66.1]	175.1 [166.4, 184.1]
	GSS	10.6 [10.1, 11.1]	65.4 [63.4, 67.4]	162.1 [149.9, 175.1]
	GLS	10.4 [9.9, 10.9]	64.4 [62.4, 66.4]	161.5 [149.1, 174.7]
	BAU + GSS	10.8 [10.3, 11.3]	65.3 [63.7, 66.9]	165.4 [154.0, 177.4]
	BAU + GLS	10.6 [10.1, 11.1]	64.7 [63.1, 66.3]	163.8 [152.3, 175.9]

Note: The ranges of future values are shown in the parenthesis.

### Drivers of changes in emissions under different fiscal stimulus scenarios

**Figure 5:**
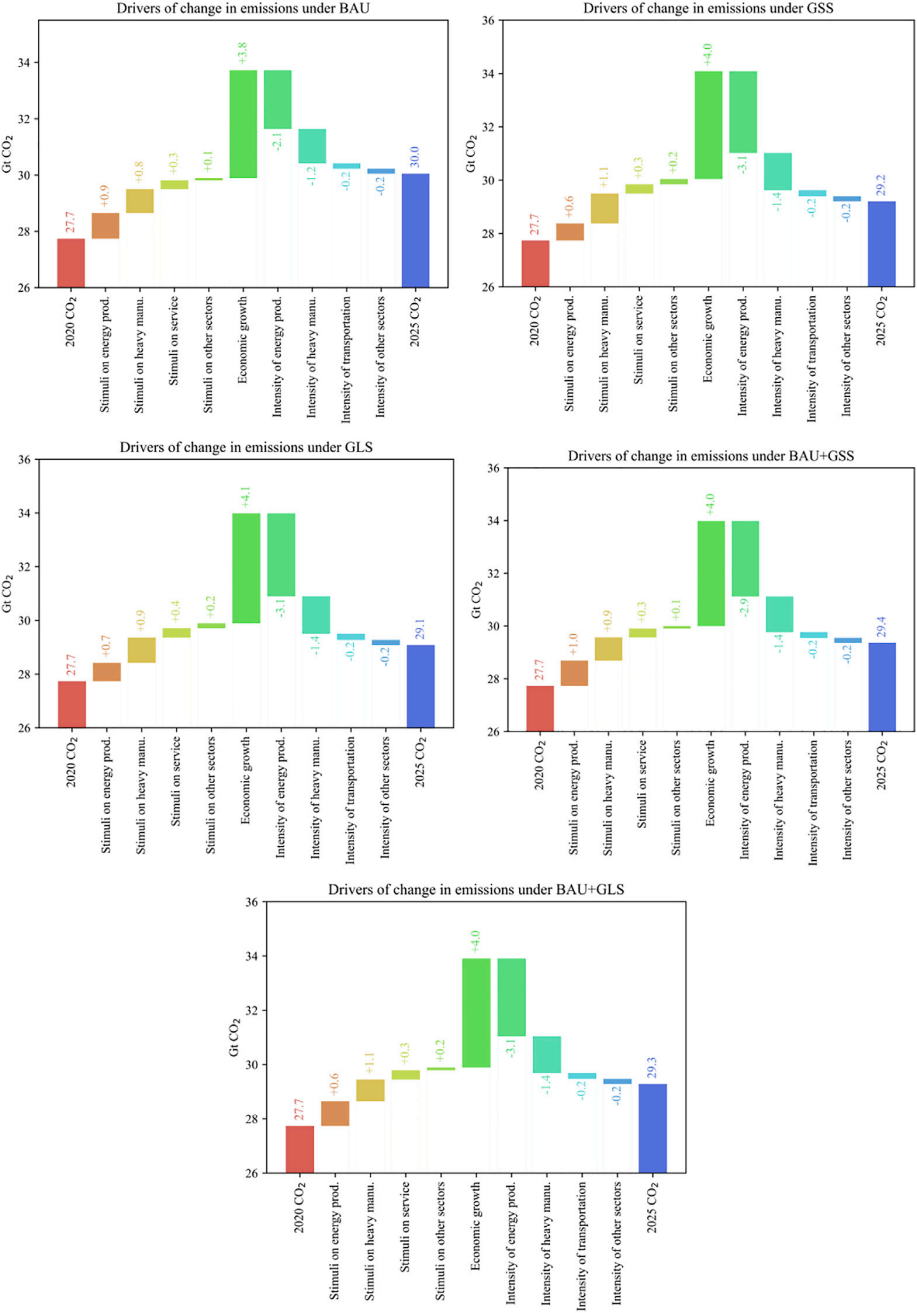
Drivers of increases in global emissions under (A) BAU, (B) GSS, (C) GLS, (D) BAU + GSS, and (E) BAU + GLS.

Figure 5 further plots the drivers of changes in emissions under five scenarios. The key insight is that the sectoral allocation of the fiscal stimulus plan will not change future emission patterns significantly, while the reduction of sectoral emission intensity will be determinant. In detail, in the absence of emission intensity change, the carbon emission difference induced by fiscal stimulus structure and economic growth between BAU and GLS scenarios is only -0.4 Gt, whereas differences in emission intensity bring about another 1.2 Gt gap. Stimuli with the same carbon intensity decline and different sectoral structure (GSS vs. GLS or BAU+GSS vs. BAU+GLS) generate similar emission outcomes. This is because the greater the increase in carbon-intensive sectors’ output is, the greater the emission-offset effect of carbon intensity decline is, as Figure 5 shows. From the sectoral perspective, the major finding is that the decarbonization of carbon-intensive sectors is dominant in future emissions patterns. Stimuli on energy production, heavy manufacturing, and service will bring the highest carbon emission growth, and the decrease in emission intensity from energy production, heavy manufacturing, and transportation sectors will bring the highest carbon emission decline. In contrast, light manufacturing, service, and high-tech sectors will be climate-friendly sectors and be responsible for smaller carbon emission increases. Agriculture and construction take up a relatively small share in China’s economy, thus exerting less prominent impacts.

## Discussion

Using multi-sector gravity model considering the nested relationship between domestic and international input-output tables, we quantitatively assess both the impacts of China’s containment policy on carbon emissions and economic output across 22 merged sectors in various countries to illustrate how the current pandemic-related carbon emission reductions and output losses are distributed along domestic and global supply chains. Compared with the emission and economic growth without pandemic, we find that China’s containment policy reduces global carbon emissions by 963.5 Mt (931.2 domestically and another 32.3 globally) and decreases global economic output by 4.8 trillion US dollars (4.4 trillion USD domestically and another 0.4 trillion USD globally), which account for 3.4% of global carbon emissions and 2.6% of world gross output, respectively. The emission shocks center in energy production and heavy manufacturing, whereas the production disruption is more dominant in service and heavy manufacturing. The inconsistent pattern between emission and production shocks reveals that the fiscal stimulus package for each sector should be designed differently in terms of scale and technical change direction. More specifically, for the carbon-intensive sectors, the pandemic should be turned into an opportunity to harness the disruption of conventional business model to redesign low-carbon policy portfolios and to optimize sectoral composition and operation standards toward decoupling between economic productivity and carbon emission impact. For the green sectors, the priority of post-COVID fiscal stimuli is to resume the return-to-normal operation and shift the economic structure toward low-carbon-based composition.

Our dynamic scenario analysis suggests that the deep decarbonization of carbon-intensive sectors is dominant for future emissions patterns. The efficiency enhancement effects rather than sectoral structure of fiscal stimulus packages are more dominant for emissions reduction. China’s current economic stimulus plan is sufficient for the 2025 emission intensity goal, but more efforts are required to achieve emission peaking in 2030 and 2°C Cancun goal. The greenness of long-term fiscal stimuli is more predominant for limiting global warming, compared to containment policies and initial fiscal stimuli in the economic recovery stage. The pandemic should thus be turned into an opportunity to harness the disruption of conventional carbon-intensive industries to redesign low-carbon policy portfolios and to optimize sectoral composition and operation standards toward decoupling between economic output and carbon emissions (or environmental impacts) (Zhang et al., 2017; Shan et al., 2018b; Wang et al., 2020; Shao et al., 2021).

Different sectors should consider different strategies to achieve eco-friendly growth. In regards to overcapacity in some heavy manufacturing and energy production sectors (especially in the production of steel and coal), firms that do not meet pollution and emission standards can be phased out, and skill development and social welfare programs should be arranged simultaneously to cope with the rising unemployment. High-tech and other service subsectors can become the focus in the economic stimulus plans and be stimulated to prevent from locking into the heavy industrialization development paths. In addition, considering that the transportation subsector is an important component of the economy, promoting low-carbon transportation systems is a key element in realizing low-carbon development. Therefore, policy portfolios should include conditional financial support for vehicles and aviation to increase the use of low-carbon or even zero-carbon fuels and investments in low-carbon urban transportation systems.

### Limitations of the study

Our model also has the following limitations or uncertainties; therefore, focusing on these aspects may provide more acute analysis of COVID-19-related emission impacts. First, the assumptions of our model on factor supply appear crude. In future works, one needs to further refine the factor and supply shocks, and thus estimate the corresponding heterogeneous economic impact. Second, China has only launched a stimulus package for the year 2021, whereas future stimulus packages are still uncertain. We assume that the future stimulus packages in 2022–2025 are scaled down to 40% of the initial one, which may not be realistic considering the unpredictable future waves of the pandemic, economic fluctuations, and trade barriers.

## STAR★Methods

### Key resources table

**Table:** 

REAGENT or RESOURCE	SOURCE	IDENTIFIER
**Deposited Data**		

MRIO tables for 2015 of 42 sectors in China	Zheng et al. (2020)	https://www.ceads.net.cn/data/input_output_tables/
Carbon emissions of each region and sector in China	Shan et al. (2018a); Shan et al. (2020)	https://www.ceads.net.cn/data/province/
International input-output tables and carbon emissions for 2014 of each country and sector	Aguiar et al. (2019)	https://www.gtap.agecon.purdue.edu/
International carbon emissions from cement production	Andrew (2018)	https://doi.org/10.5194/essd-11-1675-2019
International carbon emissions updates to 2017	IEA (2018)	https://www.oecd-ilibrary.org/energy/co2-emissions-from-fuel-combustion-2018_co2_fuel-2018-en
International carbon emissions updates to 2018	Janssens-Maenhout et al. (2019)	https://doi.org/10.5194/essd-11-959-2019
International carbon emissions updates to 2019	Friedlingstein et al. (2019)	https://doi.org/10.5194/essd-11-1783-2019
International output updates to 2019	World Bank (2020); UN (2020); IMF (2020b)	https://data.worldbank.org/indicator/NY.GDP.MKTP.CD; http://data.un.org/Data.aspx?q=table+2.4&d=SNA&f=group_code%3a204; https://www.imf.org/external/datamapper/NGDP_RPCH@WEO/OEMDC/ADVEC/WEOWORLD
Data generated by this paper (province and sector level economic shock caused by the pandemic)	This paper	Data S3

### Resource availability

#### Lead contact

Further information and requests for resources should be directed to and will be fulfilled by the lead contact, Yuli Shan y.shan@rug.nl.

#### Materials availability

This study did not generate new unique materials.

### Method details

#### Data sources

The MRIO tables for 2015 of 42 sectors in China are constructed by Zheng et al. (2020), and the associated carbon emissions of each region and sector in China are calculated by Shan et al. (2018a) and Shan et al. (2020). The international input-output tables and carbon emissions for 2014 of each country and sector are all based on the most up-to-date Version 10 of the Global Trade Analysis Project (GTAP) database (Aguiar et al., 2019). GTAP database has broad sectoral and country coverage, presenting values of intermediate products transaction between 65 sectors, the output of each sector and final consumption of commodities in 141 countries/regions. GTAP database also provides a carbon emissions matrix for each sector in each country/region, and is widely used in analyzing carbon emissions embodied in trade (Meng et al., 2018; Shan et al., 2021). We connect China’s 2015 MRIO tables and global 2014 MRIO tables, and the concordance of sectors for Chinese MRIO, Chinese sectoral carbon emissions, and GTAP database is based on the studies from Meng et al. (2018) and Mi et al. (2020), as shown in Data S5. The carbon emissions retrieved from GTAP 10 are from fossil fuel combustion, and we further include carbon emissions from cement production (Andrew, 2018), which are added to the sector of non-metallic minerals.

To calculate the total carbon emission reduction and economic output losses associated with the containment policy, we further scale up the carbon emission data by sector and country to 2017 using data from the International Energy Administration (IEA), and then to 2018 and 2019 based on the accounting from the Emissions Database for Global Atmospheric Research (EDGAR) and Global Carbon Budget 2019, respectively (IEA, 2018; Janssens-Maenhout et al., 2019; Friedlingstein et al., 2019). The data on gross output growth by sector and country in 2018 and 2019 are retrieved from the World Bank, United Nations (UN), and the International Monetary Fund (IMF) (World Bank, 2020; UN, 2020; IMF, 2020b).

#### Economic shock estimation

*Standard Leontief decomposition*. We first show how the standard Leontief decomposition works. The key point is the amount and type of intermediate input needed in the production of one-unit output, which can be traced using the linkage across countries and sectors embodied in the corresponding input-output tables. Assuming that there are *N* economic sectors in *S* regions, all gross output produced by region *s* must be used as either an intermediate or a final product at home and abroad:

$$Y_{g}^{s}=\sum_{c=1}^{N}\sum_{h=1}^{S}DI_{gc}^{h,s}Y_{c}^{h}+\sum_{c=1}^{N}\sum_{h=1}^{S^{\prime }}F_{gc}^{h^{\prime },s}$$

(Equation 1) $$\forall c,g\in N_{+}\left[1,N\right]\;\forall s,h\in N_{+}\left[1,S\right]\;\forall h^{\prime }\in N_{+}\left[1,S^{\prime }\right]$$

where $Y_{g}^{s}$, $DI_{gc}^{h,s}$, and $F_{gc}^{h^{\prime },s}$ represent the total output of sector *s* in region *g*, the direct input coefficient submatrix of the intermediate products from sector *h* in region *c* to sector *s* in region *g*, and the final demand of sector *s* in region *g* from sector *h’* in region *c*, respectively. Thus, the input-output relationship between regions and sectors can be expressed as follows (Miller and Blair, 2009):

(Equation 2) $$\left(\begin{array}{l} Y_{1}^{1}\\ \vdots \\ Y_{g}^{s}\\ \vdots \\ Y_{N}^{S} \end{array}\right)=\left(\begin{array}{lllll} DI_{11}^{1,1} & \ldots & DI_{1c}^{h,1} & \ldots & DI_{1N}^{S,1}\\ \vdots & \ddots & \vdots & \ddots & \vdots \\ DI_{g1}^{1,s} & \ldots & DI_{gc}^{h,s} & \ldots & DI_{gN}^{S,s}\\ \vdots & \ddots & \vdots & \ddots & \vdots \\ DI_{N1}^{1,S} & \ldots & DI_{Nc}^{h,S} & \ldots & DI_{NN}^{S,S} \end{array}\right)\left(\begin{array}{l} Y_{1}^{1}\\ \vdots \\ Y_{c}^{h}\\ \vdots \\ Y_{N}^{S} \end{array}\right)+\left(\begin{array}{l} F_{1}^{1}\\ \vdots \\ F_{g}^{s}\\ \vdots \\ F_{N}^{S} \end{array}\right)=DI\cdot Y+F$$

where $F_{g}^{s}$ represents the final demand of sector *s* in region *g*, which can be computed by summing up all the final demand, i.e., $F_{g}^{s}=\sum_{c=1}^{N}\sum_{h=1}^{S^{\prime }}F_{gc}^{h^{\prime },s}$. By transforming Equation (2), we can get the induced output of all economic sectors in all regions through the variation in total final demand via domestic and global supply chains. Thus, the basic multi-regional input-output (MRIO) equation can be expressed as:

(Equation 3) $$\left(\begin{array}{l} Y_{1}^{1}\\ \vdots \\ Y_{g}^{s}\\ \vdots \\ Y_{N}^{S} \end{array}\right)={\left(\begin{array}{lllll} I-DI_{11}^{11} & \cdots & -DI_{1c}^{h,1} & \cdots & -DI_{1N}^{S,1}\\ \vdots & \ddots & \vdots & \ddots & \vdots \\ -DI_{g1}^{1,s} & \cdots & I-DI_{gc}^{h,s} & \cdots & -DI_{gN}^{S,s}\\ \vdots & \ddots & \vdots & \ddots & \vdots \\ -DI_{N1}^{1,S} & \cdots & -DI_{Nc}^{h,S} & \cdots & I-DI_{NN}^{S,S} \end{array}\right)}^{-1}\left(\begin{array}{l} Y_{1}^{1}\\ \vdots \\ Y_{c}^{h}\\ \vdots \\ Y_{N}^{S} \end{array}\right)+\left(\begin{array}{l} F_{1}^{1}\\ \vdots \\ F_{g}^{s}\\ \vdots \\ F_{N}^{S} \end{array}\right)$$

After rewriting terms, we have:

(Equation 4) $$\left(\begin{array}{l} Y_{1}^{1}\\ \vdots \\ Y_{g}^{s}\\ \vdots \\ Y_{N}^{S} \end{array}\right)=\left(\begin{array}{lllll} B_{11}^{11} & \cdots & B_{1c}^{h,1} & \cdots & B_{1N}^{S,1}\\ \vdots & \ddots & \vdots & \ddots & \vdots \\ B_{g1}^{1,s} & \cdots & B_{gc}^{h,s} & \cdots & B_{gN}^{S,s}\\ \vdots & \ddots & \vdots & \ddots & \vdots \\ B_{N1}^{1,S} & \cdots & B_{Nc}^{h,S} & \cdots & B_{NN}^{S,S} \end{array}\right)\left(\begin{array}{l} F_{1}^{1}\\ \vdots \\ F_{g}^{s}\\ \vdots \\ F_{N}^{S} \end{array}\right)$$

where $B_{gc}^{h,s}$ stands for the Leontief inverse, denoting (N∗S)×(N∗S) block matrix. It represents the total requirement for gross output required by sector *s* in region *g*, when producing one-unit increase in final demand of sector *h* in region *c*. The same process can be re-conducted from Equations (1), (2), and (3), when extending to the region-level or sector-level analysis. The corresponding Leontief inverse matrices can be expressed as region-level $B_{gc}$ in Equation (5) and sector-level *B*^*h, s*^ in Equation (6), respectively:

(Equation 5) $$\left(\begin{array}{l} Y_{1}\\ \vdots \\ Y_{g}\\ \vdots \\ Y_{N} \end{array}\right)=\left(\begin{array}{lllll} B_{11} & \cdots & B_{1c} & \cdots & B_{1N}\\ \vdots & \ddots & \vdots & \ddots & \vdots \\ B_{g1} & \cdots & B_{gc} & \cdots & B_{gN}\\ \vdots & \ddots & \vdots & \ddots & \vdots \\ B_{N1} & \cdots & B_{Nc} & \cdots & B_{NN} \end{array}\right)\left(\begin{array}{l} F_{1}\\ \vdots \\ F_{g}\\ \vdots \\ F_{N} \end{array}\right)$$

(Equation 6) $$\left(\begin{array}{l} Y^{1}\\ \vdots \\ Y^{s}\\ \vdots \\ Y^{S} \end{array}\right)=\left(\begin{array}{lllll} B^{1,1} & \cdots & B^{h,1} & \cdots & B^{S,1}\\ \vdots & \ddots & \vdots & \ddots & \vdots \\ B^{1,s} & \cdots & B^{h,s} & \cdots & B^{S,s}\\ \vdots & \ddots & \vdots & \ddots & \vdots \\ B^{1,S} & \cdots & B^{h,S} & \cdots & B^{S,S} \end{array}\right)\left(\begin{array}{l} F^{1}\\ \vdots \\ F^{s}\\ \vdots \\ F^{S} \end{array}\right)$$

*International economic shocks*. Using the domestic and international input-output tables, we can calculate how the short-term economic shocks propagate from any domestic economic sectors in China’s provincial-level regions to global regions and sectors. Based on the domestic and international Leontief inverse matrix (denoted as *BD* and *BI*, respectively) derived from the corresponding input-output tables, we can calculate the international economic impact of domestic shocks as follows:

(Equation 7) $$\gamma_{gi}^{v,s}=\sum_{h}\left(BI_{gc}^{h,s}\cdot \left(\sum_{j\in c}BD_{ji}^{v,h}\cdot \gamma_{i}^{v}\cdot Y_{j}^{h}\right)/\left(\sum_{j\in c}Y_{j}^{h}\right)\right)$$

where $\gamma_{gi}^{v,s}$ reflects the impact of sector *v* in province *i* of China on sector *s* in region *g*; $\gamma_{i}^{v}$ denotes the output shock of sector *v* in province *i* when it faces $\gamma_{i}^{v}\times 100\%$ production stoppage; $BD_{ji}^{v,h}$ denotes the parameters in the domestic Leontief inverse matrix, reflecting the influence of sector *v* in province *i* on sector *h* in province *j* of China; $BI_{gc}^{h,s}$ denotes the parameters of the international Leontief inverse matrix, representing the impact of sector *h* in China (denoted by *c*) on sector *s* in region *g*; $Y_{j}^{h}$ indicates the output value of sector *h* in province *j*. Then, assuming a stable economic structure, we can calculate the short-term economic shocks of any sectoral supply or demand change in any province in China through the supply chains to each global region’s sector.

##### Economic impact model based on structural change

Under the Cobb-Douglas (C-D) setting, labor and composite intermediate goods can be used to produce intermediate goods. At the same, producers of final goods (composite intermediate goods) purchase intermediate goods as materials and follow constant elasticity of substitution (CES) production function. Eventually, final products are sold to foreign and domestic markets. Imported composite intermediates can be combined with domestic ones to produce other sectors’ composite intermediates, which can be used for consumption and produce intermediate goods. The model’s structure can be shown using the following flow chart:

**Figure:**
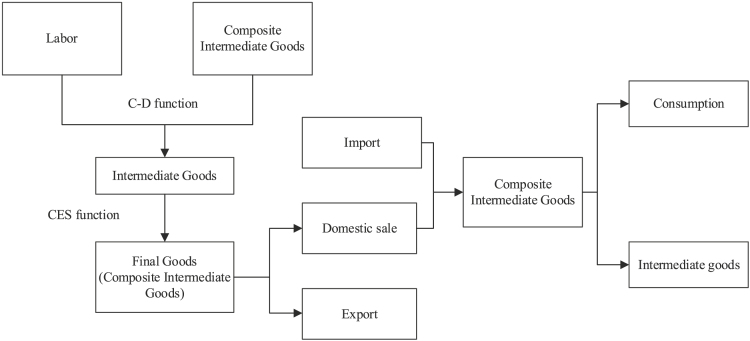

The gravity model is one of the most popular and successful frameworks in economics. Gravity model has solid theoretical foundations, which makes it particularly appropriate for counterfactual analysis, such as quantifying the effects of policy. Moreover, gravity model represents a realistic general equilibrium environment that simultaneously accommodates multiple countries and multiple sectors. As such, the gravity framework can be used to capture the possibility that markets (sectors, countries, etc.) are linked and that policy changes in one market will trigger ripple effects in the rest of the world. Finally, the gravity model has good predictive power. Empirical gravity equations of trade flows consistently deliver a remarkable fit of between 60 and 90 percent with aggregate data as well as with sectoral data for both goods and services (Yotov et al., 2016).

Compared with computable general equilibrium (CGE) models, multi-sector gravity model is more tractable and simpler for policy evaluation as it enables counterfactual analysis with fewer data and parameters through more transparent ways. Moreover, multi-sector gravity model escapes the black box denigration of traditional CGE models. In fact, CGE models include too many details and because of their complex setting, CGE models easily lose track of the mechanisms that deliver the main results. Therefore, multi-sector gravity model is more suitable for our analysis.

Integrated assessment modelling (IAM) is also a tool for integrated assessment. IAM calculates the consequences of different assumptions and interrelate many factors simultaneously, while still constrained by the quality and character of the assumptions and underlying data. Compared with IAM, multi-sector gravity model enables counterfactual analysis with fewer data and simpler settings. Although IAM enables the analysis of the effect of national fiscal policies on output, more data and parameters are required, which may not be fully available given the limited government disclosure on fiscal stimulus packages. Meanwhile, similar with CGE models, IAM’s results lack transparency, so we mainly adopt the multi-sector gravity model in this paper.

Some studies also use input-output models to study the effects of containment policies and fiscal stimuli (Shan et al., 2021). Nevertheless, economic structure is treated as stable factors in input-output model, while our economic model considers the economic structural changes. It is essential for us to do so, since the economic impact of the COVID-19 outbreak, which lasted much longer than initially anticipated, cannot be simply regarded as a short-term economic shock.

*Representative agent assumption*. We consider a global economy comprising multiple regions *(n* = 1, …, *N*) with multiple sectors *(j* = 1, …, *J*). First, we assume that the total consumption of the representative agent in region *n* can be written as:

(Equation 8) $$U\left(C_{n}\right)=\prod_{j=1}^{J}{\left(C_{n}^{j}\right)}^{s_{n}^{j}}$$

where $C_{n}^{j}$ is the final products consumption for representative individuals of sector *j* in region *n*, and $s_{n}^{j}$ denotes the consumption weight, satisfying $\sum_{j=1}^{J}s_{n}^{j}=1$. *I*_*n*_ represents agent’s total income, composed by two parts: The first is labor wage *w*_*n*_, with corresponding labor scale as *L*_*n*_, and the second is one-time transfer payments from government (tariff revenues, transfers from the rest of the world and government’s lump sum transfer to residents after subsidizing businesses).

*Intermediate and final goods production*. In this part, we mainly follow the assumptions from Caliendo and Parro (2015). Assuming that a continuum of intermediate goods in sector *j*$\left(\varpi^{j}\in \left[0,1\right]\right)$ can be produced by two types of inputs: Labor $l_{n}^{j}\left(\varpi^{j}\right)$, and the composite intermediate goods from sectors *h*, denoted by $m_{n}^{h,j}\left(\varpi^{j}\right)$. As producers of intermediate goods across countries differ in the efficiency of production, we denote $A_{n}^{j}\left(\varpi^{j}\right)$ to show their productivity. The production of intermediate goods can be written as the following C-D form:

(Equation 9) $$q_{n}^{j}\left(\varpi^{j}\right)=A_{n}^{j}\left(\varpi^{j}\right){\left[l_{n}^{j}\left(\varpi^{j}\right)\right]}^{\varepsilon_{l,n}^{j}}{\prod_{h=1}^{J}\left[m_{n}^{h,j}\left(\varpi^{j}\right)\right]}^{\varepsilon_{n}^{h,j}}$$

where $\varepsilon_{n}^{h,j}$ denotes the share of total output used to pay for the intermediate goods input from sector *h*, and $\varepsilon_{l,n}^{j}$ denotes the share for labor payment, which satisfies $\varepsilon_{l,n}^{j}+\sum_{h=1}^{J}\varepsilon_{n}^{h,j}=1$. We assume that the intermediate product market is perfectly competitive, and each price $p_{n}^{j}\left(\varpi^{j}\right)$ is at unit cost. Thus, we have $c_{n}^{j}=A_{n}^{j}\left(\varpi^{j}\right)p_{n}^{j}\left(\varpi^{j}\right)$, and unit cost function can be rewritten as:

(Equation 10) $$c_{n}^{j}=\mu^{j}{\left(w_{n}\right)}^{\varepsilon_{l,n}^{j}}\prod_{h=1}^{J}{\left(P_{n}^{h}\right)}^{\varepsilon_{n}^{h,j}}$$

where $\mu^{j}={\left(\varepsilon_{l,n}^{j}\right)}^{-\varepsilon_{l,n}^{j}}\prod_{h=1}^{J}{\left(\varepsilon_{n}^{h,j}\right)}^{-\varepsilon_{n}^{h,j}}$ is a constant, $w_{n}$ is the average wage in region *n*, $P_{n}^{h}$ is the composite intermediate price (or final product price) of sector *h* in region *n*.

We assume that each country has a final goods producer in each sector, which uses intermediate goods to produce final goods. The production function can also be written as the following CES form:

(Equation 11) $$Q_{n}^{j}={\left[\int r_{n}^{j}{\left(\varpi^{j}\right)}^{\left(\sigma^{j}-1\right)/\sigma^{j}}d\varpi^{j}\right]}^{\sigma^{j}/\left(\sigma^{j}-1\right)}$$

where $Q_{n}^{j}$ is the final product output, $r_{n}^{j}\left(\varpi^{j}\right)$ is the demand for intermediate products $\varpi^{j}$ during final production process, represents the elasticity of substitution. The final product price can be expressed as the function of intermediate price $p_{n}^{j}\left(\varpi^{j}\right)$:

(Equation 12) $$P_{n}^{j}={\left[\int p_{n}^{j}{\left(\varpi^{j}\right)}^{1-\sigma^{j}}d\varpi^{j}\right]}^{1/\left(1-\sigma^{j}\right)}$$

Thus, the demand for intermediate products satisfies $r_{n}^{j}\left(\varpi^{j}\right)={\left(\frac{p_{n}^{j}(\varpi^{j})}{P_{n}^{j}}\right)}^{-\sigma^{j}}Q_{n}^{j}$.

*International trade and economic output*. We also assume that $\kappa_{ni}^{j}$ is the bilateral trade cost of exporting from sector *j* in region *i* to region *n*, including tariff $\tau_{ni}^{j}$ and iceberg trade cost $d_{ni}^{j}$ (Iceberg trade cost $d_{ni}^{j\;2}$. Thus, the bilateral trade cost can be expressed as $\kappa_{ni}^{j}=d_{ni}^{j}\left(1+\tau_{ni}^{j}\right)$. According to Eaton and Kortum (2002), we assume that the firm productivity $A_{i}^{j}\left(\varpi^{j}\right)$ obeys Fréchet distribution; $\lambda_{i}^{j}$ is the scale parameter, $\theta^{j}$ is the shape parameter, and the cumulative probability density function is $F_{i}^{j}\left(A\right)=\exp\left(-\lambda_{i}^{j}A^{-\theta^{j}}\right)$. Then we introduce the sector subsidy variable $e_{ni}^{j}$, which denotes the subsidy for exports from region *i* to region *n* in sector *j* as a share of sector *j* output. Sectoral subsidy may change the bilateral trade cost by $\kappa_{ni}^{j}\left(1-e_{ni}^{j}\right)$ directly, according to Wang and Zhou (2021). Under complete competitive market, the final product price of sector *j* in region *n* can be expressed as:

(Equation 13) $$P_{n}^{j}=\iota^{j}{\left[\sum_{i=1}^{N}\lambda_{i}^{j}{\left(\kappa_{ni}^{j}c_{i}^{j}\left(1-e_{ni}^{j}\right)\right)}^{-\theta^{j}}\right]}^{-\frac{1}{\theta^{j}}}$$

where $\iota^{j}={\left[\Gamma \left(1+\frac{1-\sigma^{j}}{\theta^{j}}\right)\right]}^{\frac{1}{1-\sigma^{j}}}$, and $\theta^{j}>\sigma^{j}-1$. According to the CES utility function, an expression for bilateral trade flows can be obtained as:

(Equation 14) $$X_{ni}^{j}=\frac{\lambda_{i}^{j}{\left(\kappa_{ni}^{j}c_{i}^{j}\left(1-e_{ni}^{j}\right)\right)}^{-\theta^{j}}}{\Phi_{n}^{j}}X_{n}^{j}$$

where $\Phi_{n}^{j}=\sum_{f=1}^{N}\lambda_{f}^{j}{\left(\kappa_{nf}^{j}c_{f}^{j}\left(1-e_{nf}^{j}\right)\right)}^{-\theta^{j}}$. $X_{ni}^{j}$ is the total amount imported by region *n* from sector *j* in region *i*, and sectoral expenditure can be expressed as $X_{n}^{j}$, satisfying $X_{n}^{j}=\sum_{i=1}^{N}X_{ni}^{j}$. Subject to the satisfaction of the budget constraint and product market clearing, the competitive equilibrium satisfies:

(Equation 15) $$Y_{n}^{j}=\sum_{h=1}^{J}\varepsilon_{n}^{j,h}\left(1-\gamma_{n}^{j}\right)\sum_{i=1}^{N}X_{in}^{h}/\left(1+\tau_{in}^{j}\right)+s_{n}^{j}I_{n}$$

where $\gamma_{n}^{j}$ is sectoral shock. $I_{n}=w_{n}L_{n}+D_{n}+EC_{n}$ is agent’s total income, *w*_*n*_*L*_*n*_ is labor income, and *D*_*n*_ is domestic trade surpluses or deficits. *EC*_*n*_ is the government’s lump sum transfer to residents after subsidizing businesses, satisfying $EC_{n}=T_{n}^{tariff}-E_{n}$; *E*_*n*_ is government’s total subsidy to firms, satisfying $E_{n}=\sum_{j=1}^{J}\sum_{i=1}^{N}e_{ni}^{j}X_{ni}^{j}/\left(1+\tau_{ni}^{j}\right)$; $T_{n}^{tariff}$ denotes total tariff revenue, satisfying $T_{n}^{tariff}=\sum_{j=1}^{J}\sum_{i=1}^{N}\tau_{ni}^{j}X_{ni}^{j}/\left(1+\tau_{ni}^{j}\right)$.

The carbon emissions $EM_{n}^{j}$ of sector *j* in region *n* is:

(Equation 16) $$EM_{n}^{j}=et_{n}^{j}\times Y_{n}^{j}$$

where $et_{n}^{j}$ is the sectoral emission intensity of sector *j* in region *n*.

*Economic shock setting*. Our model provides a system of economic structural equations containing *N*∗*S* number of equations with *N*∗*S* unknown quantities, and there are *S* redundant equations according to the Walras’ law. As a result, given labor $\left\{L_{n}\right\}$, trade deficit $\left\{D_{n}\right\}$, productivity distribution parameter $\left\{\lambda_{n}^{j}\right\}$ and iceberg trade cost $\left\{d_{ni}^{j}\right\}$, we can obtain the output levels of sectors in each region in equilibrium under different economic shocks $\left\{\gamma_{n}^{j}\right\}$ and subsidy levels $\left\{e_{ni}^{j}\right\}$. However, solving the model requires a large number of exogenous variables and parameters. Therefore, we follow Dekle et al. (2008) and solve for equilibrium changes in prices and wages after changing $\left\{\gamma_{n}^{j},e_{ni}^{j}\right\}$. After obtaining the structural increase in output, we also considered growth projections for each country and got the combined subsidy impact (or economic shocks) of scale and sectoral structure. Then, we can get different output under different prices and wages. By comparing the changes in output levels $\left\{Y_{n}^{j}\right\}$ in two equilibrium states of no economic shocks and specific economic shocks, we can obtain the corresponding economic shock effects.

#### Measuring China’ containment policy shocks

The strict containment policy in China started in January, 2020, when traffic bans were placed on all residents, and Level 1 responses to major public health emergencies (i.e., the highest level of public health emergency response in China) are launched in provincial-level regions. Although the strictest containment policy is lifted in most provinces before April, 2020, the COVID-19-related economic downturn continued due to production disruptions, until the large-scale fiscal stimulus announced in July, 2020.

The strictness of the containment policy varies both across regions and industries, with the regional strictness determined by epidemic situation, and sectoral strictness depending on exposure risk and necessities of life. We therefore construct the counterfactual GDP growth rate without pandemic in the first half of 2020, and use the ex-post sectoral GDP growth rate minus the counterfactual GDP growth rate by sector and province without pandemic in the first half of 2020 as the proxy for $\left\{\gamma_{n}^{j}\right\}$ in Equation (7) (see Data S3 for the values for $\left\{\gamma_{n}^{j}\right\}$). The counterfactual GDP growth rate by sector and province in the first half of 2020 is primarily based on the observed GDP growth rate in the first half of 2019. Furthermore, according to OECD (2019) and UN (2019), the GDP growth rate in 2020 is expected to decline gradually by 0.2% and 0.1% compared to 2019 respectively, as Chinese economy rebalances. We therefore deduct 0.15% (i.e., the average of 0.2% and 0.1%) from observed GDP growth rate in the first half of 2019, to construct the counterfactual GDP growth rate in the first half of 2020. Carbon emission intensities in China’s sectors remain stable in the short-term lockdown because of technological bottlenecks (Liu et al., 2021).

#### Scenarios of economic stimulus plans

In order to stimulate economic recovery after the pandemic, China has launched a package of fiscal measures. According to IMF’s estimates and the Government Work Report of China, the main purpose of the package is household consumption promotion and firm tax preferences, mounting to 4.6 trillion RMB. Another 1.6 trillion of budget is allocated to the accelerated issuance of special local government bonds. The monthly report on Chinese local government bond market further specifies that the amount of special local government bond is mainly invested in infrastructure construction (65.0%), other service (26.5%), and poverty eradication (8.5%). The railway construction fund is also raised by 100 billion. The country and sector level data on economic stimulus plans in China enable us to make assumptions regarding the current scale and structure of economic stimuli. Specifically, we design five scenarios to understand the mid-term effect of China’s stimulus packages on global output and carbon emissions, including the business-as-usual (BAU), green stimulus (GSS), green lifestyle (GLS), BAU + GSS and BAU + GLS scenarios (see the following table for description and Data S4 for details). In these scenarios, the two key parameters to be determined are the scale of economic stimulus (% of GDP) and the decline in carbon intensity for each sector in China. We set $\left\{\gamma_{n}^{j}\right\}$ as the scale of economic stimulus, and use Equation (15) to simulate the impact on output. As economy recovers in 2021, the scale of sectoral economic stimulus in the later four years (i.e., 2022–2025) is assumed to be 40% of the initial scale. Next, we simulate the impact on carbon emissions using the following equation:

(Equation 17) $$EM_{n}^{j}=et_{n}^{j}\times Y_{n}^{j}$$

where *j* and *n* denote sector and region, respectively.

**Table:** 

Scenario settings of post-COVID stimuli scale and carbon intensity decline		
Scenario	Scale of economic stimulus (% of GDP)	Decline in carbon intensity
Business-as-usual Scenario (BAU)	Same as specified in China’s government report	The same as in the 14th Five-Year Plan period
Green Stimulus Scenario (GSS)	Deviate current stimulus to high-tech and service sectors	Greater decrease compared to the 14th Five-Year Plan period
Green Lifestyle Scenario (GLS)	Deviate current stimulus to light manufacturing, high-tech, and service sectors	Greater decrease compared to the 14th Five-Year Plan period
BAU + GSS	Implement fiscal stimulus as in BAU in 2021, and implement as in GSS in 2022–2025	Emission intensity decreases as in BAU in 2021, and as in GSS in 2022–2025
BAU + GLS	Implement fiscal stimulus as in BAU in 2021, and implement as in GLS in 2022–2025	Emission intensity decreases as in BAU in 2021, and as in GLS in 2022–2025

In the business-as-usual (BAU) scenario, the GDP recovers in 2021 as specified in the current scale and structure of fiscal stimulus package. The increasing investment is allocated to sectors as specified in the Government Work Report of China and monthly report on Chinese local government bond market, while the increasing investment in unspecified sectors (e.g., household consumption promotion and firm tax preferences) is allocated to all the sectors according to the pre-pandemic industrial structures. Specifically, the share of sectoral fiscal stimuli in sectoral GDP for agriculture, construction, transportation, and service in 2021 are assumed to be 4.7% (4.4%–5.0%), 21% (20.7%–21.3%), 4.9% (4.6%–5.2%), and 6% (5.7%–6.3%), respectively, while the share of sectoral fiscal stimuli in sectoral GDP for other sectors is assumed to be 4.8% (4.5%–5.1%). In the 14th Five-Year Plan period (2021–2025), China proposes the decline in emission intensity by 18%, which is the same target as in the 13th Five-Year Plan period (2016–2020). We therefore assume that the sectoral carbon intensity reduction target is the same as in the 14th Five-Year Plan period.

Green stimulus scenario assumes that the stimulus package promotes inclusive, resilient, and low-carbon post-COVID-19 economic structure. We relocate 30% of the economic stimuli initially targeted at construction to the greener sectors, with 20% relocated to high-tech sectors, and 10% relocated to service. Emission intensity decreases more significantly than the business-as-usual scenario, as green technological improvement, such as storage technology for renewable energy sources, is achieved through green-oriented financial support. The global carbon emissions per electric generation dropped by 24.2% during the pandemic due to changed diurnal circle of electric demand, and can possibly lead to the decarbonization of power system in the future (Liu et al., 2021). Therefore, we also assume China harvests the opportunity of disrupted electricity generation from conventional sources, and the emission intensity of energy production sectors decreases more significantly than other sectors.

The green lifestyle scenario further assumes that the lifestyle of Chinese household changes to a more sustainable pattern. This could occur as the interrupted transportation operation helps promote a low-carbon lifestyle of remote working and Internet conferences, and local governments promote more environmental-friendly products (e.g., electricity vehicles). In addition to the settings in the green stimulus scenario, green consumption habits are cultivated. Thus, 10% of the total stimulus of energy production, heavy manufacturing, and transportation is relocated evenly to light manufacturing and high-tech sectors. The assumptions for emission intensity are the same as green stimulus scenario.

As the epidemic poses severe and sudden shocks to economic system, primary stimulus measures emphasize the need to resume production in all walks of life. However, as the epidemic gets under control, the focus of stimuli policies may shift to other arenas, such as climate change mitigation. As suggested by Hepburn et al. (2020), to limit the global warming, it is essential for policy transitions toward green economic recovery. Therefore, we further distinguish a combination of scenarios that deals with both short-term and long-term impacts of the epidemic, i.e., BAU + GSS and BAU + GLS scenarios. In BAU + GSS scenario, the scale of fiscal stimulus and the decline in emission intensity are the same as BAU scenario in 2021, and the same as GSS scenario in 2022–2025. Similarly, in BAU + GLS scenario, the policy designs follow BAU scenario in 2021, and shift to GLS scenario in 2022–2025.

#### Monte Carlo simulation approach

The Monte Carlo simulation approach is recommended by IPCC (2006) for its flexibility and effectiveness in forecasting future carbon emission trends. As the future risks of economic downturns and the COVID-19 re-outbreak still exist in China, we introduce the Monte Carlo simulation approach to design future economic stimulus plans, which can scientifically contain uncertainties of influential factors and reveal the most possible value ranges of future carbon emissions and economic output. The procedure of Monte Carlo simulation involves three steps. First, we define pre-defined probability distributions for uncertain key input parameters, i.e., sectoral fiscal stimulus scale and emission intensity reduction rate. With respect to our study, there are some prior certainties about the most expected value as well as minimum and maximum of the range for each input parameter, but the shape of probability distribution is not precisely known. Following the suggestions of Zhang et al. (2017), we use the triangular distribution function to randomly select parameters (see Data S4 for the information of the upper bounds, lower bounds, and median values of the parameters). Second, we conduct multiple simulations by randomly sampling the parameter space according to the pre-defined probability distributions. Totally, 1000 Monte Carlo simulations are carried out for each parameter to generate more accurate results. Third, the ranges, as well as median values of the potential carbon emissions and economic output are presented in the results section.

## Data Availability

• This paper analyzes existing, publicly available data which are listed in the key resources table. The data generated by our analysis can be found in Data S3. • Code for the analysis was written in Matlab and is available from the lead contact upon request. • Any additional information required to reanalyze the data reported in this paper is available from the lead contact upon request.

## Supplemental information

Data S1. Categorization of sectors used in this study

Data S2. Categorization of world regions

Data S3. Province and sector level economic shock caused by the pandemic

Data S4. Assumptions for sectoral fiscal stimuli and emission intensity reduction

Data S5. Concordance of sectors for Chinese MRIO, Chinese sectoral CO2 emissions and GTAP database
